# An Oxygen Vacancy Memristor Ruled by Electron Correlations

**DOI:** 10.1002/advs.202201753

**Published:** 2022-07-28

**Authors:** Vincent Humbert, Ralph El Hage, Guillaume Krieger, Gabriel Sanchez‐Santolino, Anke Sander, Sophie Collin, Juan Trastoy, Javier Briatico, Jacobo Santamaria, Daniele Preziosi, Javier E. Villegas

**Affiliations:** ^1^ Unité Mixte de Physique CNRS Thales Université Paris‐Saclay Palaiseau 91767 France; ^2^ CNRS IPCMS UMR 7504 Université de Strasbourg Strasbourg 67034 France; ^3^ Grupo de Física de Materiales Complejos Dpt. Física de Materiales Universidad Complutense de Madrid Madrid 28040 Spain

**Keywords:** memristors, neuromorphic systems, nickelates, strongly correlated electrons

## Abstract

Resistive switching effects offer new opportunities in the field of conventional memories as well as in the booming area of neuromorphic computing. Here the authors demonstrate memristive switching effects produced by a redox‐driven oxygen exchange in tunnel junctions based on NdNiO_3_, a strongly correlated electron system characterized by the presence of a metal‐to‐insulator transition (MIT). Strikingly, a strong interplay exists between the MIT and the redox mechanism, which on the one hand modifies the MIT itself, and on the other hand radically affects the tunnel resistance switching and the resistance states' lifetime. That results in a very unique temperature behavior and endows the junctions with multiple degrees of freedom. The obtained results bring up fundamental questions on the interplay between electronic correlations and the creation and mobility of oxygen vacancies in nickelates, opening a new avenue toward mimicking neuromorphic functions by exploiting the electric‐field control of correlated states.

## Graphical abstract

A reversible oxygen exchange at the interface between an amorphous metal (Mo_80_Si_20_) and a strongly correlated oxide (NdNiO_3_, NNO) yields strong resistive switching effects. Interestingly the metal‐to‐insulator transition characteristic of NNO interplays with that redox mechanism, leading to a very unique behavior that opens new avenues for mimicking neuromorphic functions by exploiting the electric‐field control of correlated states.

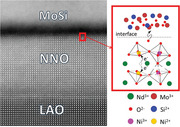

## 1 Introduction

Resistive switching effects have attracted increasing attention during the last decade. Beyond their fundamental interest, they provide the basis for ultrafast, low energy consumption memories.^[^ ^1^ ^]^ They can be realized by exploiting a variety of mechanisms, e.g., spin torques in magnetic tunnel junctions,^[^ ^2^ ^]^ phase transitions in strongly correlated materials,^[^ ^3^ ^]^ ionic motion in oxide capacitors,^[^ ^4^, ^5^ ^]^ polarization switching in ferroelectric tunnel junctions,^[^ ^6^ ^]^ or reversible redox reactions at metal/oxide tunnel junctions.^[^ ^7^ ^]^ The common denominator of those different systems is that a reversible switching between a high resistance (OFF) and a low resistance (ON) state is triggered by application of electrical stimuli (e.g., voltage or current pulses). One of the key figures of merit is the ratio between the electrical resistance *R* in the OFF and ON states, which in the case of electron tunneling devices is conventionally termed electroresistance^[^ ^6^, ^7^, ^8^ ^]^ *E*_r_ ≡ *R*_OFF_/*R*_ON_ = *G*_ON_/*G*_OFF_ (with *G* the electrical conductance) and can be as high as *E*_r_ ∼ 10^7^ at room temperature^[^ ^9^ ^]^ and *E*_r_ ∼ 10^9^ at low temperatures.^[^ ^10^ ^]^ In addition, some of the above‐mentioned systems show memristive behavior. This is characterized by the fact that the switching between ON/OFF states (and often across a continuum of intermediate resistance states^[^ ^11^ ^]^) is controlled not only by the size^[^ ^12^ ^]^ but also by the history^[^ ^13^ ^]^ of electrical stimuli. This functionality constitutes a crucial ingredient for the emergent field of neuromorphic computing^[^ ^14^, ^15^, ^16^, ^17^ ^]^ because, depending on the resistance states’ lifetime, memristors can mimic the behavior of either synapses or neurons.^[^ ^13^, ^18^ ^]^

The family of rare‐earth (RE) nickelates RENiO_3_ attracts increasing attention in the above context.^[^ ^8^, ^19^, ^20^, ^21^ ^]^ Those strongly correlated materials present a rich phase diagram,^[^ ^22^, ^23^ ^]^ in which the interplay between various degrees of freedom can be tuned through multiple knobs (temperature, rare‐earth substitution,^[^ ^23^ ^]^ doping,^[^ ^24^, ^25^ ^]^ strain,^[^ ^26^, ^27^ ^]^ electric field,^[^ ^28^, ^29^ ^]^ light,^[^ ^27^, ^30^, ^31^ ^]^ stoichiometry^[^ ^20^, ^32^ ^]^) to access very different ground states: metallic‐to‐insulating,^[^ ^33^, ^34^ ^]^ magnetic, or even superconducting.^[^ ^35^ ^]^ Here, we show that these properties can be exploited to enrich resistive switching effects via an unexpected, tantalizing effect: the strong electronic correlations come to dominate the motion of oxygen ions that, driven by electric fields, produce giant resistive switching effects in the present experiment.

The investigated devices are junctions between NdNiO_3_ (NNO) and a metal (the amorphous alloy Mo_80_Si_20_, hereafter MoSi). In this simple structure, a tunnel barrier is naturally formed at these materials’ interface due to a spontaneous redox reaction that oxidizes MoSi at the expense of reducing the interfacial NNO, producing a local predominance of Ni^2+^ (as opposed to Ni^3+^ away from the interface). That leads to the junctions’ virgin state, which shows high resistance. The oxygen exchange between the two sides of the junction can be reversed electrochemically, upon application of a voltage pulse. This thins down the tunnel barrier (NNO layer with predominance of Ni^2+^) and leads to the low resistance (ON) state, yielding a giant tunnel electroresistance *E*_r_ up to ≈10^4^ (i.e. ≈10^6^ %). The application of negative/positive voltage pulses allows repeated switching across a quasi‐continuum of intermediate resistance states between the low (ON) and high (OFF) resistance levels, which endows the junctions with memristive behavior. Interestingly, this redox switching mechanism dramatically interplays with the strong electron correlations underlying the NNO's characteristic metal‐to‐insulator transition (MIT) at $T_{\mathrm{MIT}}∼70\;K$. This interplay has two manifestations. On the one hand, the resistive switching into the OFF state is accompanied by a shift to lower temperatures of the MIT as probed by the electron tunneling measurements. That effect is the fingerprint of Ni valence modulation at the MoSi/NNO interface. On the other hand, and conversely, the MIT greatly affects the switching behavior: the size of *E*_r_, the magnitude of the voltages required to produce the ON/OFF switching, and the lifetime of the resistance levels are markedly different at both sides of the MIT. This can be explained within a scenario in which the strong electronic correlations developing in the insulating phase ultimately inhibit the creation/annihilation and mobility of oxygen vacancies within the NNO film. In addition to their fundamental breadth, and despite the simplicity of the studied devices from the fabrication standpoint, our findings pave the way to memristive devices that exploit the strongly correlated nature of the nickelates (and particularly the MIT), thereby providing tunability of key parameters, such as *E*_r_ and the lifetime of the resistance states.

## 2 Fabrication and Characterization

### 2.1 Nickelate Thin Film

**Figure 1:**
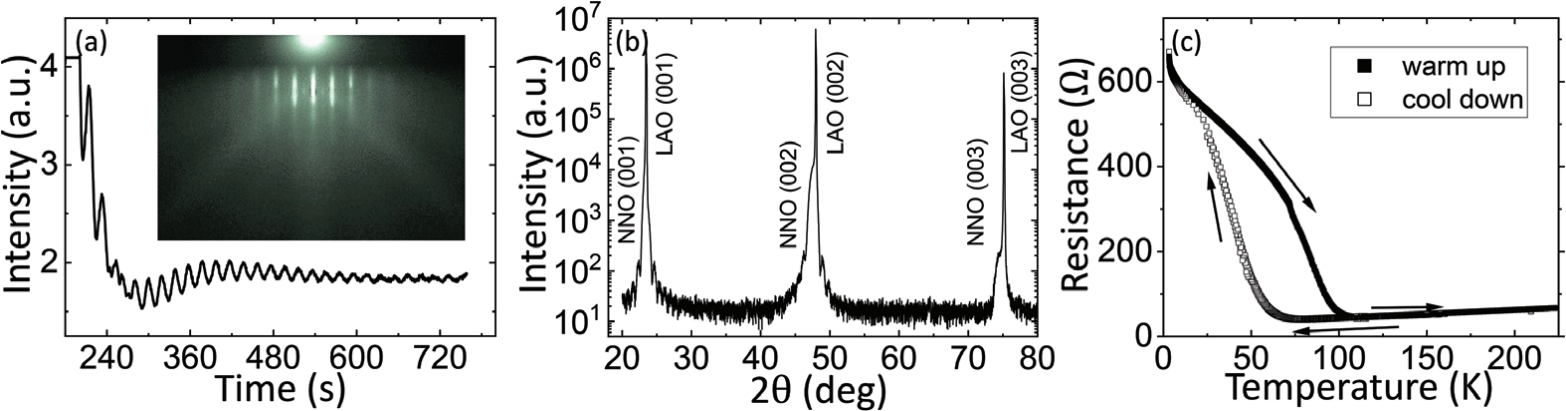
Characterization of the NdNiO_3_ film. a) RHEED taken during the growth of the NdNiO_3_ film. The oscillations and the RHEED patterns allow to monitor a layer‐by‐layer growth of the nickelate on the substrate and its crystalline structure. b) XRD of the film demonstrating growth along the 001 axis, following the crystalline structure of the LAO substrate. c) Four‐probe measurement of the temperature‐dependent NdNiO_3_ film resistance during cooling down (hollow) and the warming up (solid).

12 nm thick NdNiO_3_ films were synthesized by pulsed laser deposition onto LaAlO_3_ (LAO) single crystals as described in the Experimental Section. Reflection high‐energy electron diffraction (RHEED) oscillations (**Figure** **1**a) and X‐ray diffraction (XRD) patterns (Figure 1b) indicate a layer‐by‐layer epitaxial film growth following the [001] axis of the LAO substrate. Bulk NNO exhibits an orthorhombic crystal structure with a lattice parameter of ≈0.381 nm in the pseudo‐cubic notation. Thus, the NNO grows onto LAO single crystals (pseudo‐cubic lattice parameter of 0.379 nm), under a small (−0.52%) compressive strain that produces an elongation of the out‐of‐plane lattice parameter (0.383 nm) as inferred from the XRD data. This is just as expected from the LAO‐induced compressive strain according to a Poisson ratio of 0.25–0.3, which suggests that the NNO films present a low concentration of native oxygen vacancies as demonstrated in similar pulsed laser deposition‐grown NNO thin films.^[^ ^25^ ^]^

Figure 1c shows the typical NNO film's resistance as a function of the temperature, which was measured using a conventional four‐probe configuration. At high temperature, the film shows metallic behavior. Upon cooling, a first‐order metal‐to‐insulator phase transition takes place, as evidenced by the sharp resistance increase of more than one order of magnitude at around $T_{\mathrm{MIT}}∼70K$. Expectedly, this MIT is accompanied by a paramagnetic to antiferromagnetic transition.^[^ ^36^ ^]^ Upon temperature increase, the MIT is observed at a higher $T_{\mathrm{MIT}}\;∼\;100\;K$. This hysteretic behavior is as expected for a first‐order transition,^[^ ^36^ ^]^ and denotes the coexistence of insulating and metallic domains allowing for temperature‐dependent percolation paths,^[^ ^37^ ^]^ similarly to other MIT materials.^[^ ^38^, ^39^ ^]^

### 2.2 Nickelate‐Based Junctions

#### 2.2.1 Native Properties of the NNO/MoSi Interface

**Figure 2:**
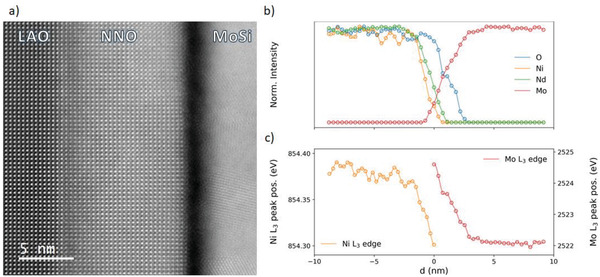
Scanning transmission electron microscopy characterization of the NdNiO_3_–MoSi interface. a) Atomic resolution Z‐contrast STEM image of a NdNiO_3_ (NNO)–MoSi bilayer grown onto a LaAlO_3_ substrate (LAO). b) Normalized integrated intensity profiles of the O K (blue), Ni L_2,3_ (orange), Nd M_4,5_ (green), and Mo L_2,3_ (red) edges across the NdNiO_3_/MoSi interface. c) Ni L_3_ and Mo L_3_ peak positions depict chemical shifts at the interface due to variations in their respective oxidation states.

As we detail below, the NNO/MoSi junctions are fabricated by masked sputtering deposition of MoSi on NNO. The interface, structural and chemical properties of NNO/MoSi bilayers were investigated by combining scanning transmission electron microscopy (STEM) and electron energy loss spectroscopy (EELS). Atomic resolution high‐angle annular dark field images (**Figure** **2**a) prove the highly epitaxial growth of the nickelate on the substrate (LaAlO_3_). The overlaying MoSi is amorphous near the interface and eventually presents small (nanometric) crystallites away from it. The EELS‐integrated intensity profiles (Figure 2b) show a sharp drop (≈1 nm wide) of the Ni and Nd intensity at the NNO/MoSi interface (yellow and green lines in that Figure). However, the oxygen signal (blue line) extends 2–3 nm into the MoSi, clearly indicating the interfacial oxidation of this material. The Mo and Ni oxidation states at the interface can indeed be inferred from the shifts of the respective L_2,3_ edges. The Mo L_3_ edge shifts by 2.5 eV (from 2522 to 2524.5 eV, red line in Figure 2c). According to literature data,^[^ ^40^ ^]^ that shift denotes a change from a metallic state to a 6+ oxidation state, consistent with the formation of MoO_3_. Concerning Ni, the Ni L_3_ edge (roughly at 854 eV) shifts from 854.4 (away from the interface) to 854.3 eV at the interface (orange line in Figure 2c). This can be interpreted in terms of a reduction in the oxidation state from 3+ inside the nickelate layer towards a 2+ at the interface.^[^ ^41^ ^]^ In summary, the microscopy analysis of the as‐grown samples indicates that oxygen migrates from NNO into MoSi, yielding interfacial layers of oxygen‐deficient NNO and oxidized MoSi. This result is not surprising: redox reactions are ubiquitous at complex oxides interfaces, and are the origin of a variety of phenomena^[^ ^42^ ^]^ including resistance switching in superconducting cuprate/metal interfaces.^[^ ^7^ ^]^ In the present case, if we consider that the reduction potential of Ni (*E*_0 (Ni)_ = 0.36 V for Ni^3+^ to Ni^2+^)^[^ ^43^ ^]^ is higher than that of Mo and Si (respectively, *E*_0 (Mo)_ = − 0.2 V for Mo^3+^ to Mo^2+^ and *E*_0 (Si)_ = − 0.8 V for Si^2+^ to Si^[^ ^44^ ^]^), one indeed expects that the interfacial MoSi spontaneously oxidizes at the expense of reducing the interfacial NNO. As we discuss in what follows, such redox reaction can be reversed upon application of sufficiently strong voltage pulses, which leads to the switching of the conductance across the NNO/MoSi interface.

#### 2.2.2 Junctions Fabrication and Measurement Setup

**Figure 3:**
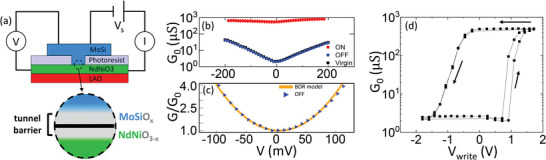
Measurement configuration and electrical characterization of junctions. a) Scheme of the MoSi/NiNdO_3_ vertical junctions studied. The NiNdO_3_ film deposited on top of a LaAlO_3_ substrate is covered by photoresist, in which photolithographed openings define the junction area. A MoSi films (capped with Au) is then deposited. Measurements are performed by applying a voltage bias *V*_s_ with a K2450 sourcemeter and measuring the circulating current *I*. The voltage drop across the junction *V* is measured with a voltmeter. The oxygen exchange at the interface yields to oxidized MoSi and oxygen‐depleted NNO layer that behaves as a tunnel barrier. b) Differential conductance measurement performed at 100 K after applying writing voltages *V*_write_ = 1.71 V (ON state, red) and *V*_write_ = − 1.8 V (OFF state, blue). The virgin state is shown in black. c) Fit to the BDR model of the *I*(*V*) in the OFF state at 100 K to the BDR model. d) Remnant zero‐bias differential conductance after application of *V*_write_ following the cycle indicated by the arrows in steps Δ*V*_write_ ∼ 0.2 V. An electroresistance in excess of 100 (over two orders‐of‐magnitude resistance switching) is observed. Two cycles measured subsequently appear superposed.

A schematic of the junctions fabricated for the transport experiments is shown in **Figure** **3**a. Starting from a 12 nm thick film of NNO on LAO, we performed photolithography to define openings in a photoresist layer spin‐coated on the NNO. The openings have a typical area of few squared microns. The junctions are created by depositing amorphous MoSi directly on the NNO surface across those openings, using room‐temperature sputtering. MoSi is subsequently covered in situ by gold to protect it, and to facilitate electrical bonding. Further details of the fabrication process are described elsewhere.^[^ ^7^ ^]^ Electrical contacts were made by bonding gold wires directly on both the NNO film and the MoSi, as represented in Figure 3a. Notice that two leads are attached to the NNO and two others to the top of the MoSi/Au electrode to allow for four‐probe measurements. The conductance of the devices is measured following the scheme in Figure 3a, i.e., by applying a voltage *V*_s_ between a pair of leads and measuring the current *I*. In order to precisely measure the actual voltage drop across the junction *V*, without sensing the voltage drop across the wires and the NNO layer, a voltmeter was connected to the other pair of leads as shown in Figure 3a. Unless otherwise specified, all of the voltages referred to hereafter correspond to the voltage drop across the junction *V* measured in that fashion. The differential conductance *G*(*V*) ≡ *dI*/*dV* is numerically calculated from *I*–*V* measurements. The measurements performed in this geometry probe the conductance across the MoSi/NNO interface where, as shown in Figure 2, oxidized MoSi and oxygen‐deficient NNO layers are formed which behave as a tunneling barrier. To set the junction in the different resistance states, the voltage across the junction is ramped up to the desired “writing” voltage *V*_write_ and then ramped back to zero which, as explained further below, changes the thickness of the tunneling barrier. For the measurements shown in the manuscript, the “writing” ramp duration is in the range of tens of seconds. However, as shown in Figure S6 in the Supporting Information, application of much shorter (≈ ms) single *V*_write_ pulses produces exactly the same effects. The *I*–*V* characteristic in the remnant state (after application of *V*_write_), is measured without exceeding *V* = 200 mV, which is low enough to avoid any further change in the junction's resistance state.

#### 2.2.3 Memristive Behavior

Figure 3b shows *G*(*V*) at 100 K after cooling down from room temperature, that is above the MIT of NNO, for different junction's states. *G*(*V*) in the virgin state, as measured before applying any *V*_write_ to the junction, is shown in black. That is almost identical to *G*(*V*) in the OFF state (blue curve in Figure 3b, which is set after application of *V*_write_ = − 1.8 *V*). This implies that the OFF state is the native, equilibrium state of the junction. *G*(*V*) in the OFF state can be fitted using the Brinkman–Dynes–Rowell (BDR) model^[^ ^45^ ^]^ up to *V* = 100 mV, as shown in Figure 3c. This model describes tunnel conductance as a function of the bias voltage by considering electron tunneling across a trapezoidal potential barrier.^[^ ^45^ ^]^ A good agreement between model and experiments is obtained for a 5 nm tunnel barrier width, with a 0.25 eV barrier height and a barrier asymmetry of ≈60% (see the Experimental Section). Consistently, the estimated 5 nm barrier thickness is comparable to the combined thickness of the oxygen‐depleted NNO and oxidized MoSi layers observed in the STEM experiments (Figure 2). The conductance in the ON state (red curve in Figure 3b), set upon application of *V*_write_ = 1.71 V, is more than two orders of magnitude higher than in the OFF state. In the ON state, the fitting of *G*(*V*) to the BDR model is not satisfactory, which may be due to the dominance of inelastic tunneling as in the case of cuprate‐based junctions.^[^ ^7^ ^]^ The conductance of the studied junctions scales with their size, as shown in Figure S3 in the Supporting Information. Similarly as in superconducting cuprate‐based junctions^[^ ^7^ ^]^ and ferroelectric tunnel junctions,^[^ ^46^ ^]^ the OFF state conductance scales with the junction area—which rules out conduction through local defects and is consistent with electron tunneling over the junction area^[^ ^47^ ^]^, while the ON state scales with the junction's perimeter. The latter suggests that the conductance switching is produced by homogenous changes over the junctions’ periphery. This is explained^[^ ^7^, ^46^, ^48^ ^]^ by the fact that the applied electric field is stronger over the edges of the junction, favoring the local activation of the switching mechanism (here, the motion of oxygen ions).

The memristive behavior is demonstrated in Figure 3d, which displays the remnant zero‐bias differential conductance *G*_0_ = *G*(0), set by the application of different *V*_write_ (*x*‐axis) at 100 K. *V*_write_ is cycled from negative to positive and vice versa as indicated by the arrows. A hysteretic switching is observed between two “plateaus,” corresponding to the OFF and ON states. The switching from OFF to ON sets in around $V_{\mathrm{write}}∼1V$, and is completed for *V*_write_ ≥ 1.7 V. Conversely, the switching from ON into OFF starts around $V_{\mathrm{write}}∼-0.4V$, and is completed at *V*_write_ ≤ − 1.7 V. There is thus a clear asymmetry, which indicates that the switching from the ON into the OFF state is easier than the opposite process, as reported for other redox systems^[^ ^7^ ^]^, and also for ferroelectric tunnel junctions.^[^ ^8^, ^11^ ^]^ In Figure 3d, the electroresistance reaches $E_{r}∼3\;\times \;10^{2}$ and, as shown in Figure S4 in the Supporting Information, smaller area junctions can reach even a larger value $E_{r}\;∼\;10^{4}$ due to the different scaling of the ON/OFF conductance level with the junctions’ size.^[^ ^7^, ^46^, ^48^ ^]^ Crucially, the switching between ON and OFF occurs gradually, across intermediate states which are nonvolatile at temperatures below 150 K. The number of intermediate levels can be controlled by varying the size of the voltage steps Δ*V*_write_ across the cycle (compare Figure 3d to Figure 5a) and also via the sequence of applied *V*_write_ (see Figure S4, Supporting Information). As demonstrated by Figure S5 in the Supporting Information, the junctions can be repeatedly switched at least hundreds of times with no significant degradation of *E*_r_, which remains well in excess of 10^2^.

#### 2.2.4 Temperature Dependence

**Figure 4:**
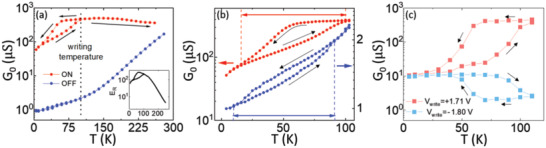
Temperature behavior of the junctions. a) Zero‐bias differential conductance as a function of temperature, which is changed following the cycle 100 K → 5 K → 250 K (see arrows). The conductance is measured after writing the ON (red) or OFF (blue) states, respectively, with *V*_write_ = +1.71 V and *V*_write_ = −1.80 V at a fixed *T*_write_ = 100 K. Inset: Corresponding electroresistance as a function of temperature. b) Close up on the same curves as in (a), allowing to see the MIT signatures in the temperature‐dependent junctions’ conductance. Notice that the MIT temperature is shifted down by ≈20 K in the OFF state. c) Remnant zero‐bias conductance states measured after applying fixed *V*_write_ = +1.71 V (magenta) and *V*_write_ = −1.80 V (cyan) at variable temperature *T*_write_, which is changed following the cycle 120 K → 5 K → 120 K as indicated by the arrows. The conductance is also measured at *T*_write_.

**Figure** **4**a shows the temperature‐dependent zero‐bias conductance, *G*_0_(*T*), for both ON and OFF states. Each of these states was set by applying *V*_write_ ≃ 1.71 V (ON) and *V*_write_ ≃ −1.8 V (OFF) at a fixed *T* = 100 K after cooling from room temperature, i.e., they were set at a temperature at which the NNO electrode is in its metallic phase. After the application of the corresponding *V*_write_ at *T* = 100 K, *I*–*V* measurements of the remnant state were performed every 10 K, following the sequence 100 K → 5 K → 250 K, which allowed us to extract the *G*_0_(*T*) curves displayed in Figure 4a.

In the ON state (red curve), we observe a strongly hysteretic behavior reminiscent of the NNO's film *R*(*T*) (see Figure 1c). In particular, upon decreasing the temperature from 100 K, the conductance is nearly constant down to *T*_MIT_. Below this temperature, the conductance drops over one order of magnitude across the MIT of the NNO electrode. This trend is reversed as the temperature is ramped up. However, similarly as in Figure 1c the transition temperature is shifted to higher temperatures, which results in the hysteresis characteristic of the MIT. Finally, from *T* = 125 K to *T* = 250 K, we observe a gradual decrease of the conductance. As demonstrated further below, the later behavior is due to a natural relaxation from the ON towards the OFF state.

In the OFF state (blue curve), the background temperature dependence is relatively strong over the entire temperature range: the conductance decreases by about two orders of magnitude between 250 K and 10 K. The hysteretic behavior is also present, but it is weaker, and only visible in a zoom of the curve—see Figure 4b. The temperature dependence of the conductance in the ON and OFF states results in a strong temperature‐dependent *E*_r_, which also displays the hysteretic behavior characteristic of the MIT in NNO, as shown in the inset of Figure 4a. To the best of our knowledge, this hysteretic effect has not been observed in other resistive switching devices based on nickelates (such as ferroelectric tunnel junctions^[^ ^8^ ^]^), and implies that the junction's conductance reflects the electronic properties of the NNO electrode.^[^ ^49^ ^]^

A careful examination of the zoomed conductance versus temperature curves (Figure 4b), reveals yet another very interesting effect: the hysteresis characteristic of the MIT is shifted to lower temperatures in the OFF state as compared to the ON state. In average, that shift is of nearly 20 K. This suggests that the mechanism leading to the resistive switching is changing the *T*_MIT_ of the interfacial NNO.

Strikingly, the resistive switching behavior is strongly dependent on the temperature at which the “writing” voltage pulses *V*_write_ are applied. This is shown in Figure 4c, which displays the remnant conductance measured after applying opposite writing voltage pulses [*V*_write_ ≃ + 1.71 V (magenta) and *V*_write_ ≃ − 1.8 V (cyan)], at varying temperature *T*_write_. This is changed in steps following the cycle indicated by the arrows. The fixed values of *V*_write_, respectively, correspond to the voltages required to set the ON and OFF states at *T*_write_ = 100 K (see the cycle in Figure 3d). It is important to stress that we have ensured that the applied *V*_write_ is the same for all *T*_write_ by measuring the voltage drop across the junction with a voltmeter as shown in Figure 3a. The amplitude of the switching achieved with the fixed *V*_write_ is the largest when *T*_write_ is within the range in which the NNO is in its metallic phase—here the electroresistance is nearly constant $E_{r}\;∼\;3\;\times \;10^{2}$. However, it gradually decreases as *T*_write_ is decreased and the NNO undergoes the transition into the insulating phase, until there is no switching ($E_{r}\;∼\;1$) for *T*_write_ < 20 K. A large *E*_r_ is recovered when *T*_write_ is increased again above 100 K, and the NNO goes back to its metallic phase (once again, the thermal hysteresis characteristic of the MIT is reflected in the measurement). In summary, the *V*_write_ that produces a full ON/OFF switching in the NNO's metallic phase is unable to do so in the insulating one. This indicates that the NNO's insulating phase hinders the resistive switching mechanism.

**Figure 5:**
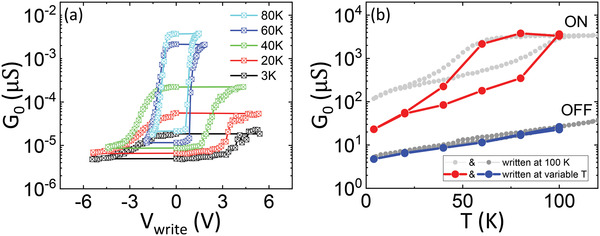
Switchability as a function of temperature. a) Zero‐bias differential conductance versus *V*_write_ cycles, measured at different *T*_write_ (see the legend) that is decreased from 80 to 3 K. The writing voltage is cycled in steps Δ*V*_write_ which are chosen to be smaller at high temperatures (≈0.05 V at 80 K) than at low ones (≈0.15 V at 3 K) to stabilize many intermediate states. b) In color, remnant zero‐bias conductance as a function of temperature after switching the junction in the ON (red) or OFF state (blue) at variable temperatures *T_write_*, which requires varying *V*_write_ as shown in (a). The temperature is varied following the cycle 100 K → 3 K → 100 K. In the background, shown in gray shades, the temperature‐dependent zero‐bias conductance measured after switching at a fixed *T*_write_ is shown.

Further insights supporting the above conclusion can be drawn from the measurements shown in **Figures** **5**a,b. Figure 5a shows switching cycles *G*_0_(*V*_write_) for different *T*_write_ (see the legend). One can see that, as the temperature is decreased, the cycles become wider and the resistance switching amplitude diminishes. That is, upon decreasing *T*_write_, there is a significant increase of the *V*_write_ required to produce the ON/OFF switching and the achieved resistance variation gets smaller. One can also see that the increase of the switching *V*_write_ is more pronounced in the OFF → ON branch of the cycle (further details are provided in Figure S2, Supporting Information). Also, the ON conductance level is the most dramatically reduced as *T*_write_ is decreased, while the OFF conductance is essentially unaffected. All of the above suggest that the onset of the NNO's insulating state upon decreasing temperature dramatically hinders the switching into the ON state. This is also evident in Figure 5b, which displays the conductance level *G*_0_ achieved in both ON (red) and OFF (blue) states in the case of variable *T*_write_, to be compared with the temperature‐dependent ON/OFF conductance levels measured after writing at a fixed *T*_write_ = 100 K (gray colored data). Once again, one can see that the OFF‐state conductance is similar regardless of *T*_write_, while the ON‐state level is significantly lower if *T*_write_ lies within the range in which NNO is in its insulating phase.

#### 2.2.5 Lifetime and Relaxation of Resistance States

**Figure 6:**
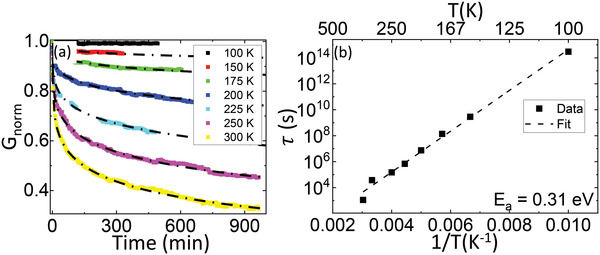
Relaxation of the ON state. a) Normalized zero‐bias conductance as a function of time at the temperatures shown in the legend (different colors), after having set junction in the ON state at *T*_write_ = 100 K. Dashed lines correspond to stretch exponential fits. b) Evolution of the characteristic time as a function of the temperature. The dotted line is a linear fit allowing to extract the activation energy *E*_a_.

As mentioned above, the OFF state corresponds to the equilibrium state of the junctions, while the ON state is metastable and relaxes toward the OFF state over a time scale that depends on the temperature. This is shown in **Figure** **6**a, which displays the evolution of the conductance as a function of time after the junction has been set in the ON state. In order to quantify the relaxation dynamics, we carried out the same measurement protocol for all temperatures: first, we systematically preconditioned the junction at *T* = 100 K, by initially switching the junction into the OFF and then into the ON state. After that, we ramped the temperature to a desired *T* and, once *T* was stable, recorded the conductance *G*_0_ as a function of time *t*. The data have been normalized as *G*_norm_(*t*) ≡ (*G*_0_(*t*) − *G*_OFF_)/(*G*_ON_ − *G*_OFF_), so that *G*_norm_ = 1 in the ON state and *G*_norm_ = 0 in the OFF state. At *T* = 100 K (black data), the relaxation is very slow—the conductance drops by less than 1% over 10 h—making the ON state virtually nonvolatile within the experimental time scale. However, a faster relaxation is observed as soon as the temperature reaches 150 K (red data), and the relaxation rate keeps increasing as the temperature increases. For all the temperatures, the conductance decays from the ON level following a stretched exponential law^[^ ^50^ ^]^ as typically found in disordered systems that present a distribution of relaxation times:

(1) $$G_{\mathrm{norm}}=e^{-{\left(\frac{t}{\tau }\right)}^{\beta }},$$

where 1/*τ* is the global relaxation rate, and 0 < *β* < 1 is a constant. In our case, we can fit the conductance relaxation (dashed lines in Figure 6a) using a unique *β* = 0.25 for all the temperatures. Thus, the only temperature‐dependent fitting parameter is the relaxation rate 1/*τ*. Figure 6b demonstrates that the relaxation rate follows an Arrhenius law $\tau \propto e^{E_{a}/k_{B}T}$, where *E_a_* is the activation energy. From this analysis, we deduce that the relaxation is governed by an activation energy *E*_a_ = 0.31 eV, as shown by the linear fit in Figure 6b.

## 3 Discussion

All the described observations can be consistently explained by considering that the spontaneous interfacial redox reaction leading to oxygen exchange between MoSi and NNO (see Figure 2) can be reversed upon application of *V*_write_. Interestingly, the electronic correlations that drive the MIT, interplay with that redox process, leading to the unusual behavior described in this paper. Such an interplay is discussed in what follows.

Let us start by recalling some key aspects of the NNO's electronic properties, which strongly depend on its oxygen content. Fully stoichiometric NNO, in which the oxidation state of Ni is nominally 3+, displays a *d* ^8^L metallic ground state at high temperature, and a low temperature MIT that is driven by transformation of the *d* ^8^L into bond‐disproportionate *d* ^8^L^0^ and *d* ^8^L^2^ states, with alternate population of the ligand hole L states.^[^ ^51^ ^]^ The low‐temperature phase corresponds to a weak insulator (small gap *E*_g_ ∼ 0.5 eV), and excitations are from the weakly correlated oxygen band. A slight deoxygenation leads to moderate replacement of Ni^3+^ by Ni^2+^, which produces a decrease of *T*_MIT_ and an increase of overall resistivity.^[^ ^24^ ^]^ Upon further oxygen depletion, the accumulation of oxygen vacancies leads to a more substantial substitution of Ni^3+^ by Ni^2+^ and a strongly insulating state even at room temperature.^[^ ^20^ ^]^ Electron localization close to Ni^2+^ sites results from the filling of the oxygen ligand holes, what enhances the effect of the Coulomb repulsion in Ni^2+^ *d* ^8^ states. In addition, a crystal field modification occurs due to the reduced oxygen coordination, which further promotes localization, all of it leading to a large charge gap that can reach up to 2 eV, as measured in similar nickelates.^[^ ^20^ ^]^

**Figure 7:**
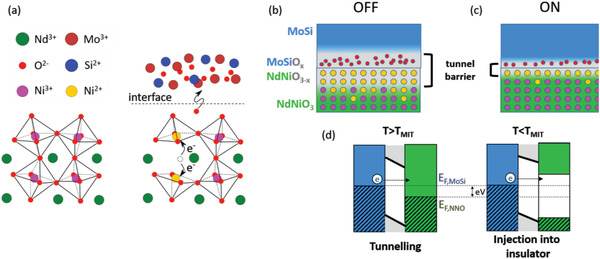
Resistive switching model. a) Structure of the NNO (orthorhombic) and MoSi (amorphous) close to the interface. The OFF state is produced by oxygen migration from the NNO into MoSi, which leads to Ni^+2^ states at the interface. In the OFF state, the insulating barrier (oxidized MoSi and oxygen‐depleted, Ni^+2^‐rich layers) is thicker and the presence of Ni^+2^ extends beyond the interface, leading to a locally depressed *T*_MIT_. c) In the ON state, produced upon application of a *V*_write_ that partially reverses the redox reaction, the Ni^+2^ and oxidized MoSi layer are much thinner, leading to higher conductance and higher *T*_MIT_. d) Sketch of the band structure across the interface. Above *T*_MIT_, an electron at the Fermi level can tunnel from MoSi into NNO. As the temperature is lowered below *T*_MIT_, aninsulating gap opens in the NNO‐ In the low‐bias regime of the conductance measurements electrons are injected into that gap, which explains the large conductance decrease below *T*_MIT_.

We can now consider electrochemical arguments to explain the conductance switching based on changes in the interfacial oxidation states produced upon application of *V*_write_. As demonstrated in Figure 2, and analogously as in cuprate/MoSi junctions,^[^ ^7^ ^]^ a spontaneous redox reaction at the interface produces an accumulation of oxygen vacancies in NNO, which leads to a local valence change^[^ ^20^, ^43^ ^]^ from Ni^3+^ to Ni^2+^ (see sketch of the NNO structure in **Figure** **7**a), and thus^[^ ^20^ ^]^ to a highly insulating interfacial NNO layer. On the MoSi side, oxidation leads to an insulating state.^[^ ^7^ ^]^ As a result, in the ground (OFF) state of the junctions, the interface (see sketch in Figure 7b) consists of highly insulating oxygen‐deficient NNO and oxidized MoSi layers, which constitute an electron tunneling barrier—whose effective thickness is of ≈ 5 nm according to the BDR analysis (Figure 3b) and the STEM/EELS experiments (Figure 2). In the NNO metallic state (*T* > *T*_MIT_), the redox reaction can be reversed by application of a *V*_write_ exceeding both the difference between reduction potentials (Δ*E*_0_ ≃ 0.56 − 1.16 V) and the barrier for oxygen diffusion. This re‐oxidizes the interfacial NNO, and consequently reduces the interfacial MoSi, thus thinning the tunnel barrier and leading to a more conductive interface (ON state, see sketch in Figure 7c). This state is metastable, and the switching back to the equilibrium (OFF) state can be achieved in two ways. The first possibility is to activate the redox reaction that oxidizes MoSi (reduces NNO) via the application of a reversed *V*_write_ < − 0.4 V. Notice the asymmetry of the onset |*V*_write_| (OFF→ON)∼1V versus |*V*_write_| (ON→OFF)∼0.4V in Figure 3d. This stems from the fact that the latter process is spontaneous from the electrochemical point of view. Thus, its activation only requires overcoming the barriers for oxygen diffusion—which determines the dynamics of the reaction. Indeed, as shown by the relaxation measurements in Figure 6a, if temperature is high enough, the ON → OFF switching occurs spontaneously over a measurable time scale, due to thermal activation over the diffusion barrier. Notice that, consistently, the energy scale $E_{a\;}\;∼\;0.3\;$eV extracted from the analysis in Figure 6b is comparable to the threshold |*V*_write_| < 0.4 V required to switch from the ON into the OFF state in the temperature range *T* > *T*_MIT_ where NNO is metallic. The stretched exponential behavior indicates that a distribution of energy barriers for ion migration exists. This can be understood if one considers that the oxygen mobility decreases as the oxygen‐deficient NNO is gradually replenished and the interfacial density of oxygen vacancies diminishes, which is consistent with the long tail observed across the resistance switching (see the hysteresis loop of Figure 3d).

The correlation between the MIT in the NNO electrode and the salient features of the temperature‐dependent tunneling conductance—in particular the strong conductance decrease across the MIT and hysteresis observed in Figures 4a,b—can be readily understood based on the junction's scheme shown in Figure 7d. In the metallic state of NNO (*T* > *T*_MIT_), electron tunneling occurs between two metallic electrodes (MoSi and NNO). However, below *T*_MIT_, an insulating gap opens in the NNO.^[^ ^22^, ^52^ ^]^ Thus, within the low bias *V* range used for the measurements, the current is not anymore injected into a metal, but into the gapped state of a charge‐ordered insulator (see right sketch in Figure 7d). This explains the strong conductance decrease observed across the MIT (Figure 4a), which is particularly strong in the ON state. In the OFF state, the conductance decrease associated with the MIT is also visible but it is less pronounced, due to the dominant effect of the thicker, strongly insulating (Ni^2+^ rich) interfacial layer, which presents a charge gap that can be as large as 2 eV.^[^ ^20^ ^]^ Very interestingly, such sensitivity of the tunneling conductance to the MIT allows detecting that the *T*_MIT_ is shifted by ≈ 20 K to lower temperatures upon switching from the ON into the OFF state (Figure 4b). This shift indicates that, in the OFF state, oxygen depletion extends further from the interface (see scheme in Figure 7b) and so, in addition to a first few nm in which Ni^2+^ is predominant (tunnel barrier), a significant Ni^2+^ presence exists deeper into the NNO which locally depresses *T*_MIT_,^[^ ^24^ ^]^ in good agreement with the microscopy observations (Figure 2). Notice that the above discussion focuses on changes across the interface (*z* axis) without the consideration of the lateral length scales (*xy* plane). In this sense, it is worthwhile noting that, as pointed out earlier and shown in Figure S3 in the Supporting Information, the ON state's conductance scales with the junction's perimeter. This implies that the oxygen exchange leading to NNO doping upon switching into the ON state occurs homogenously over the junction's periphery, and not over its entire area. That is as expected^[^ ^7^ ^]^ because the electric field is stronger there.^[^ ^46^ ^]^ Thus, one has to bear in mind that the switching between the two different states sketched in Figures 7b,c occurs over the junction periphery, and not over the rest of the junction area, which remains in the state sketched in Figure 7b. It is precisely the fact that the state in Figure 7c is set first in the periphery, thereby opening a highly conducting shunting channel, which prevents the remaining of the junction area to switch into the same state.

Let us finally discuss the fact that, strikingly, the ON/OFF switching becomes increasingly harder when cooling across the MIT. This is evidenced by the increasing switching voltages and the radical weakening of the resistance switching amplitude observed as *T*_write_ is decreased below the MIT of the NNO (Figures 4c and 5). Notice that the OFF → ON switching process is the most dramatically affected: the writing voltage increases much more drastically than for the ON → OFF one (see Figure 5a and Figure S2 of the Supporting Information). Also, only the ON conductance level is severely scaled down as *T*_write_ is decreased (see Figure 5b). Such unique behavior is fundamentally interesting; particularly, because it is explained by the onset of electronic correlations in NNO. As discussed above (Figure 7b,c), the resistance switching is ultimately linked to the creation/annihilation of oxygen vacancies in NNO, which, respectively, lead to Ni^2+^/Ni^3+^ states. Thus, the much weaker switchability at *T* < *T*_MIT_ indicates that, in the NNO's insulating state, oxygen vacancies become strongly localized. The key to understand this is to consider that oxygen vacancies carry a doping field, and thus, that the annihilation of an oxygen vacancy involves the switching of the excess (doping) electron so that Ni^2+^ becomes Ni^3+^. While doping electrons are delocalized in the metallic state, in the insulating state they are strongly localized at the Ni^2+^ site close to the vacancy. This is because, in the insulating state, electron switching requires excitation over the very large (2 eV) local energy gap^[^ ^20^ ^]^ created by Coulomb repulsion. Therefore, oxygen vacancies can be annihilated only if the energy necessary to create a Coulomb excitation is available, which in our case is supplied by the external voltage. This explains why the OFF → ON switching, which implies oxygen transport from the MoSi into the NNO—and consequently the annihilation of oxygen vacancies—requires increasing writing voltages upon entering the insulating state. Notice that the reverse process (ON→OFF switching) results from a Ni^3+^ to Ni^2+^ valence conversion, which requires electron transfer to the Ni^3+^ in order to close a ligand hole (*d* ^8^L→ *d* ^8^). This process is also hindered by the electronic correlations in the insulating phase, but can occur at a lower energy cost due to the smaller gap of the Ni^3+^ phase and the weaker correlations in the oxygen band. In summary, in the strongly correlated insulating phase of NNO, the energy cost of the charge excitation required to create/annihilate oxygen vacancies naturally explains why resistive switching effects are hindered—and the asymmetry between OFF → ON and ON → OFF grows—upon decreasing temperatures. Conversely, switching is much easier in the metallic phase of the NNO because of the large density of states at the Fermi level. This provides the delocalized electrons required for the creation or annihilation of vacancies, and thereby lifts the limitations imposed by the electronic correlations. Thus, interestingly, the same electronic correlations that underlie the MIT ultimately rule the redox reaction that leads to the resistance switching.

## 4 Conclusion

In summary, we fabricated junctions with an amorphous MoSi alloy on top of a nickelate NdNiO_3_ thin film. These junctions show large tunnel electroresistance and memristive^[^ ^11^ ^]^ behavior. The mechanism underpinning these effects is oxygen migration across the interface, which is driven by reversible redox reaction between the NNO and MoSi layers. The high‐conductance (ON) state of the junction is metastable and relaxes toward the low‐conductance (OFF) ground‐state over a temperature‐dependent time scale. This is extremely long at low temperatures, making the resistance states virtually nonvolatile below 100 K, and gradually shortens as temperature is increased.

The central result of our study is that the MIT characteristic of the NNO electrode interplays with the redox mechanism, yielding different regimes in which the resistance states are either “frozen” or become switchable depending on whether the NNO is in its insulating or metallic phase. This brings about interesting fundamental questions about how strong electronic correlations can condition the creation/annihilation of charged defects in this type of materials. Despite the extreme structural simplicity of the studied junctions—just a metal/oxide interface—the very unique phenomenology they present opens the door to prospective applications that, beyond resistive switching devices and memristors, include the electrical manipulation of strongly correlated oxides ground state and the electric‐field control of topotactic reactions,^[^ ^35^, ^53^ ^]^ which in the particular case of nickelate thin films^[^ ^35^ ^]^ can stabilize a superconducting ground‐state. Finally, the different relaxation behavior of ON (metastable) and OFF (stable) states of the junctions may enable synaptic‐like (nonvolatile states) or neuron‐like (relaxing states) functionalities depending on the temperature. To materialize that, potentiation/depression and leaky‐integrate‐and‐fire behaviors need to be explored,^[^ ^54^ ^]^ what will be accomplished in the future.

## 5 Experimental Section

NdNiO_3_ were synthesized by PLD onto LaAlO_3_ (LAO) single crystals by ablating a (43%) single phase ceramic target (Toshima Manufacturing Co.) with a KrF excimer laser at a repetition rate of 2 Hz and with a fluence of ≈4 J cm^−^^2^. LaAlO_3_ (LAO) single crystals were annealed in air for 10 h at 1200 °C to show a single‐phase surface termination. After growth, the NNO//LAO samples were cooled down at a rate of 5 °C min^−1^ in the same oxygen partial pressure. Single phase and epitaxial NNO thin films (see Figure 1a) were obtained in a high oxidizing atmosphere (0.15 mbar of O_2_) and at a temperature of 675 °C. After the growth, the films were stocked under nitrogen atmosphere until processing them by standard lithography techniques.

a‐MoSi DC sputtering was performed using a base vacuum pressure of 5e‐8 mbar. Deposition of 100 nm thick MoSi occurred under pressure of 2.5e‐3 mbar of Ar. The sputtering power was of 50 W using a commercial target of Mo_80_Si_20_. The deposition rate was of 1.3 A s^−1^. After deposition, MoSi was covered with 10 nm of gold to protect the surface and ease gold wire bonding. The resistivity of the MoSi deposited on Si/SiO_2_ substrate was of 4 × 10^−7^ Ω m.

The STEM experiments were carried out in JEOL JEM ARM 200cF systems and the EELS measurements were made with a GATAN Quantum spectrometer.

The resistance of the MoSi/NdNiO_3_ junctions was measured using a Keithley 2450, applying a voltage output while measuring the current passing through the circuit. The voltage drop at the border of the junction was measured by a Keithley2182a nanovoltmeter to perform 4 probe measurement. For the preparation of the resistance states shown in the main text, the K2450 ramped the voltage by using about 20 steps to reach the desired writing voltage *V*_write_ before stepping down to zero. Each voltage step was applied for 1 s; consequently, the writing time was ≈20 s. It was verified that an identical resistance switching was obtained if instead a single *V*_write_ pulse as short as 20 ms was applied (see Figure S6, Supporting Information).

The quantitative analysis of the tunnel barrier of this junction used the BDR model.^[^ ^45^ ^]^ In this model, the conductance was depended on the applied voltage through the following expression

(2) $$\frac{G\left(V\right)}{G\left(0\right)}=1-\left(\frac{A_{0}\Delta \phi }{16\phi^{\frac{3}{2}}}\right)\mathrm{eV}+\left(\frac{9A_{0}^{2}}{128\phi }\right){\left(\mathrm{eV}\right)}^{2}$$

where Δ*φ* is the barrier asymmetry, *φ* is the average barrier height, and $A_{0}=\frac{4{\left(2m\right)}^{\frac{1}{2}}d}{3\bar{h}}\;$ where *m* is the electron mass and *d* is the barrier thickness. The BDR fit presented in Figure 3c was obtained by using the fitting parameters of a 5 ± 0.5 nm thick insulating barrier between NdNiO_3_ and MoSi. This barrier was found to be of 0.25 ± 0.02 eV with an asymmetry of 60%. While not needed to fit the data, this asymmetry allowed to well describe the asymmetry of the conductance as a function of the bias voltage.

## Conflict of Interest

The authors declare no conflict of interest.

## Supporting information

Supporting Information

## Data Availability

The data that support the findings of this study are available from the corresponding author upon reasonable request.
