# Supplementary material for: An Oxygen Vacancy Memristor Ruled by Electron Correlations
Source: Adv Sci (Weinh). 2022 Jul 28;9(27):2201753. doi: 10.1002/advs.202201753 (PMC9507366; doi:10.1002/advs.202201753)
Supplement: Supplementary file 1 — Supporting Information [file ADVS-9-2201753-s001.pdf]

Supporting Information

An oxygen vacancy memristor ruled by electron correlations

Vincent Humbert [1], Ralph El Hage [1], Guillaume Krieger [2], Anke Sander[1], Sophie Collin[1], Juan Trastoy[1], Javier Briatico[1], Jacobo Santamaria[3], Daniele Preziosi[2,], and Javier E. Villegas[1,♦]*

E-mails: *daniele.preziosi@ipcms.unistra.fr, ♦javier.villegas@cnrs-thales.fr

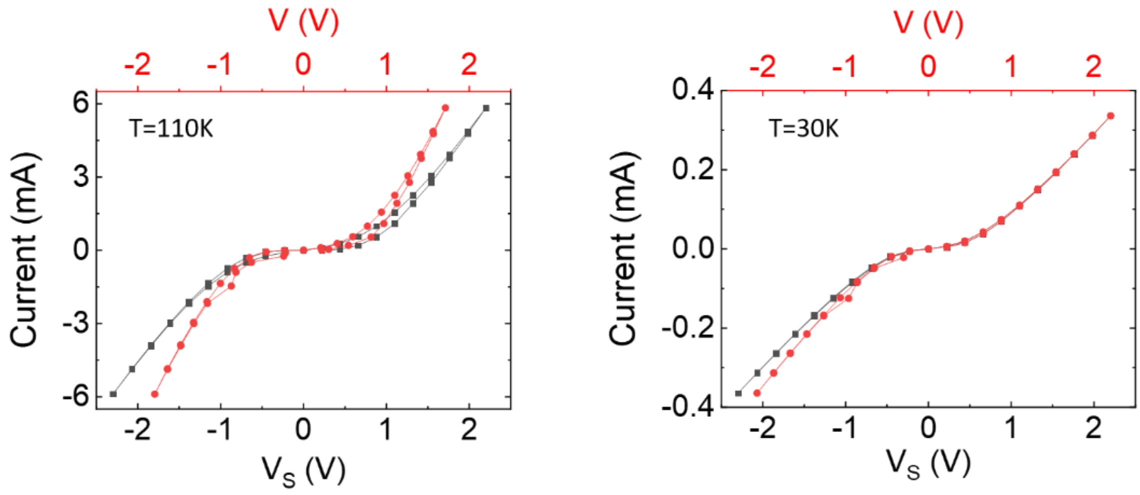

Figure S1. I(V) curves recorded during the application of V_{write} (maximum/minimum applied voltage). In black, we show the voltage applied by the source V_S, and in red the voltage measured across the junction V. This shows that the junction is the most resistive element of the circuit, even in the NNO's insulating state (measurement at 30 K). All the voltages discussed in the main text are always the voltages measured across the junction, V.

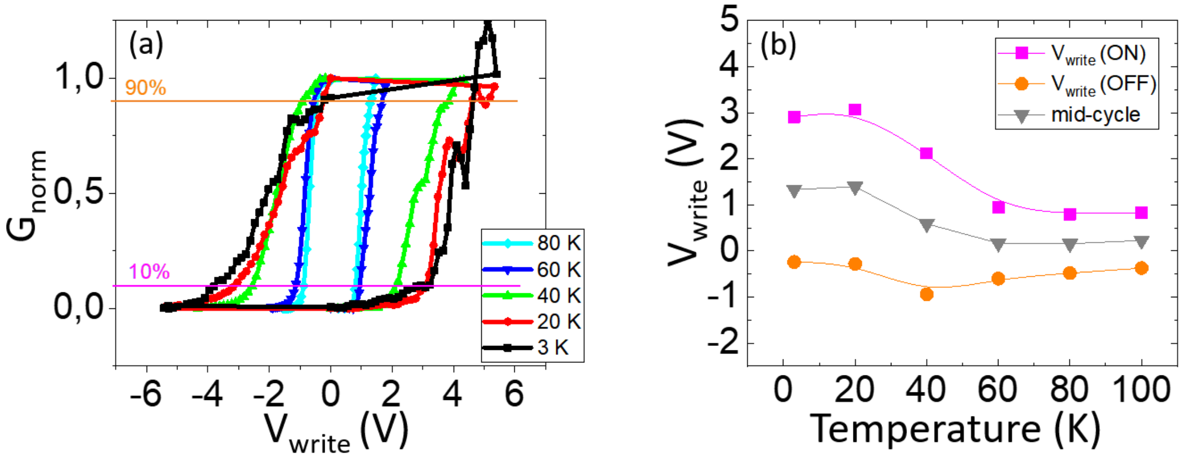

Figure S2. (a) Zero-bias differential conductance versus V_{write}, measured at different T_{write} (see legend) that is varied by cooling from 100 K to 3 K. The data shown here is the same as in Figure 4a, although normalized such that "1" corresponds to the highest ON state and "0" to the OFF state. This allows defining the switching voltages using the criterion indicated by the horizontal lines: 90% of the ON level for the ON $\rightarrow$ OFF branch [$V_{write}(OFF)$] and 10% above the OFF level for OFF $\rightarrow$ ON switching [$V_{write}(ON)$]. (b) ON/OFF switching voltages (as defined by the above criteria) and mid-cycle voltage (defined as $^1/_2\,[V_{write}(ON) - V_{write}(OFF)]$ as a function of temperature. One can see that, upon decreasing temperature across the MTI, $V_{write}(ON)$, increases drastically, thus increasing the cycle asymmetry as temperature is lowered.

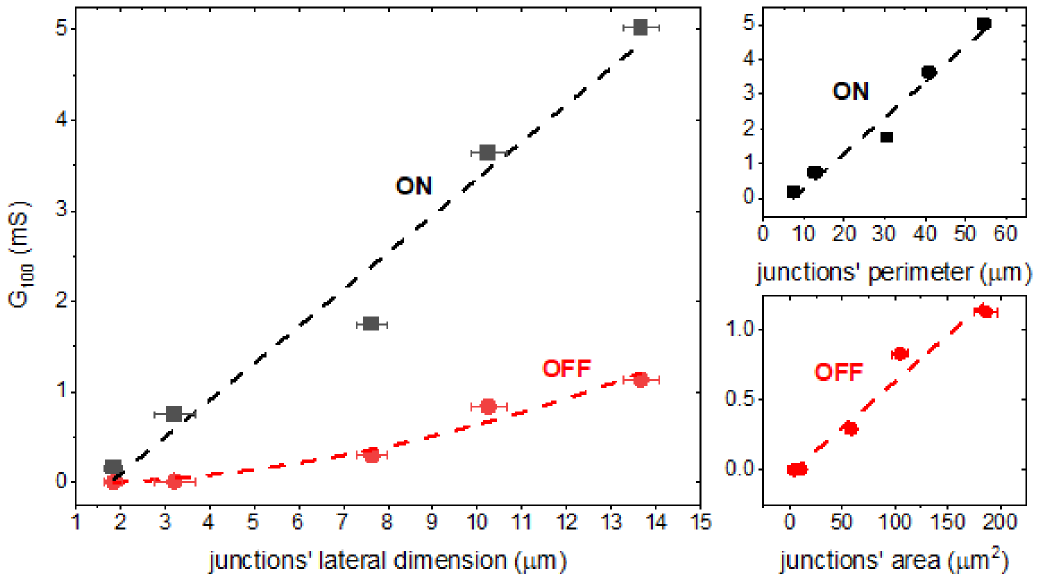

Fig. S3: Conductance in the ON (black) and OFF (red) state measured at V_{read} =100 mV and T=100 K for a series of square junctions with different sizes. The main panel (left) shows the conductance as a function of the junctions' lateral dimension (square side). In the ON state, the conductance is roughly proportional to that dimension (the dashed line is the best linear fit). At variance, in the OFF state, the conductance increases with the squared dimension (the dashed line is the best fit to a parabola). The representation of the same data in the right panels further supports that the ON-state conductance is proportional to the junction's perimeter (top panel) and the OFF-state conductance is proportional to the junctions' area (bottom panel). Here the dashed lines are the best linear fits. Overall, the behavior is similar to that observed in MoSi/$YBa_2Cu_3O_7$ junctions [V. Rouco et al. Nature Communications volume 11, 658 (2020)]. The scaling in the OFF state rules out conduction through local defects, such as filaments or pinholes [*Mater. Today* **11,** 28–36 (2008)], and is consistent with homogeneous electron tunneling over the junction area. On the other hand, the conductance in the ON state scales with the junctions' perimeter. This implies that the resistance switching occurs *homogeneously* over the junctions' periphery. That is explained [see e.g. *APL* **109,** 232902 (2016)] by the fact that the electric field produced by the application of V_{write} is strongest over the junction's edges, locally favoring the activation of the switching mechanism (here, the motion of oxygen ions).

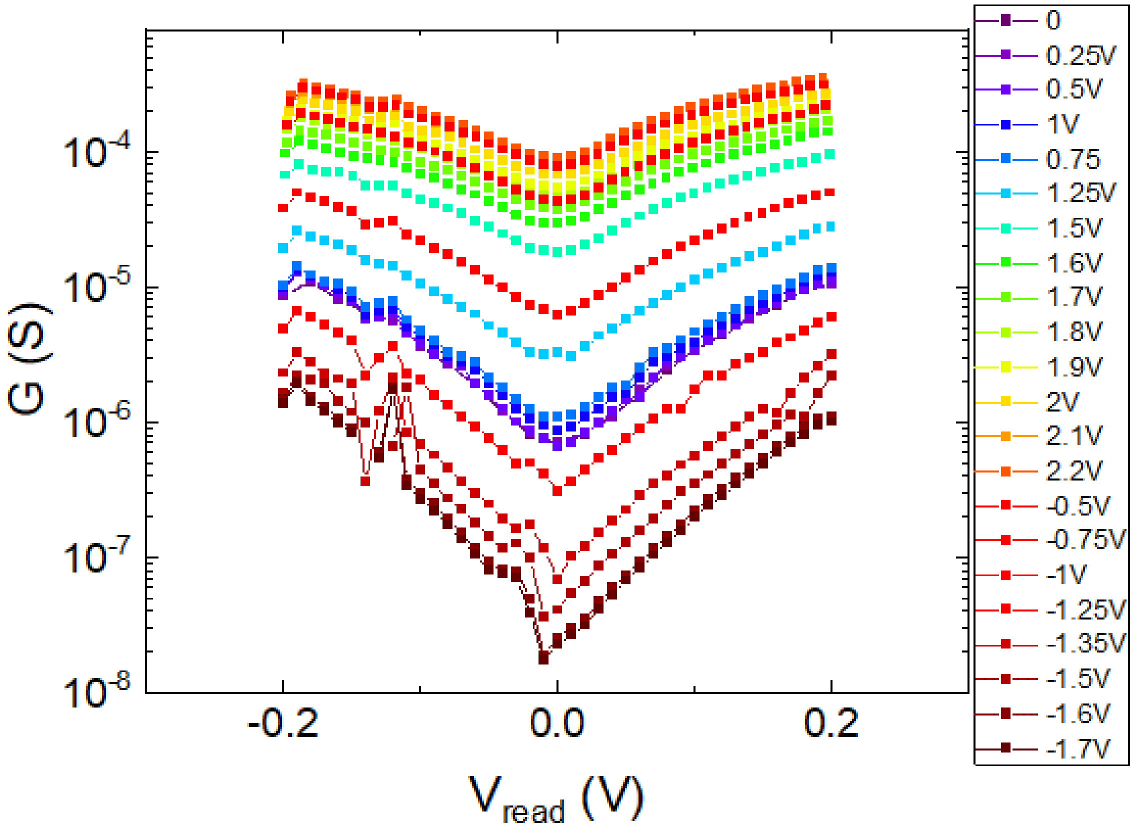

Fig. S4: Differential conductance *vs.* bias $G(V_{read})$ measured in the remnant state after application of different writing voltages V_{write} following the sequence indicated in the legend (0V → 2.2 V → -1.7 V). The writing voltages are applied (and the subsequent conductance measurements carried out) at T = 100 K. The ON and OFF conductance levels (respectively upper and lower curves) are four orders of magnitude apart. Notice that many intermediate states can be set by the V_{write} sequence. The junction's lateral size is here 1.854 ± 0.2 μm.

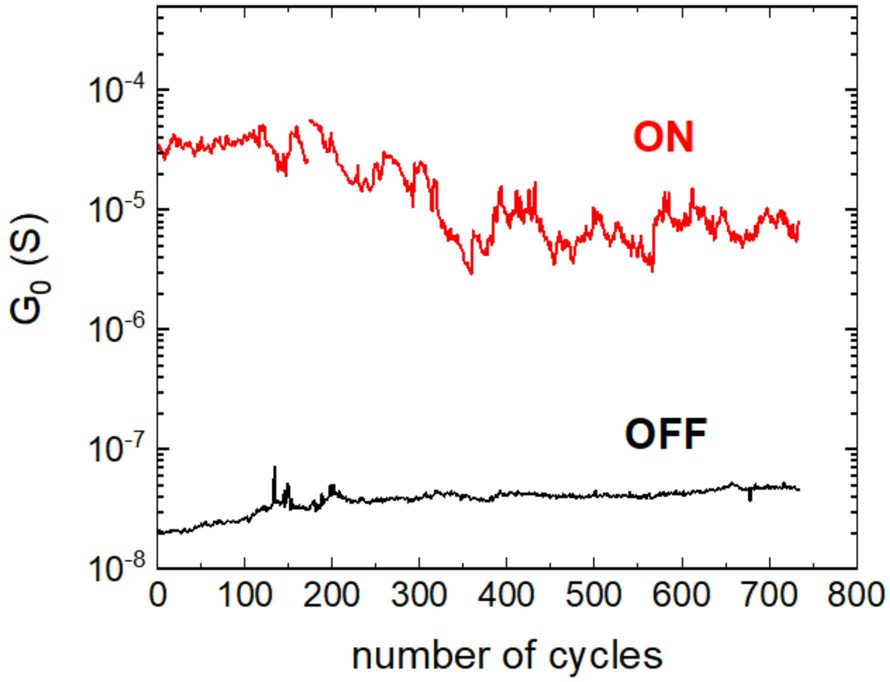

Fig. S5: Zero-bias conductance level in the ON (red) and OFF (states) as a function of the number of switching cycles. For these measurements, each cycle consist of the application of 1 second long writing pulses of amplitude V_{write}=1.9 V (switching into ON) and V_{write}=-1.55 V (switching into OFF), each pulse followed by a low-bias measurement of the remnant conductance G_0 . The cycles are repeated up to 750 times to monitor the endurance of the junctions. Despite the ON/OFF ratio being slightly reduced after the first few hundred switching cycles, the characteristic E_r in excess of 10^4 % is preserved and remains unchanged upon further cycling.

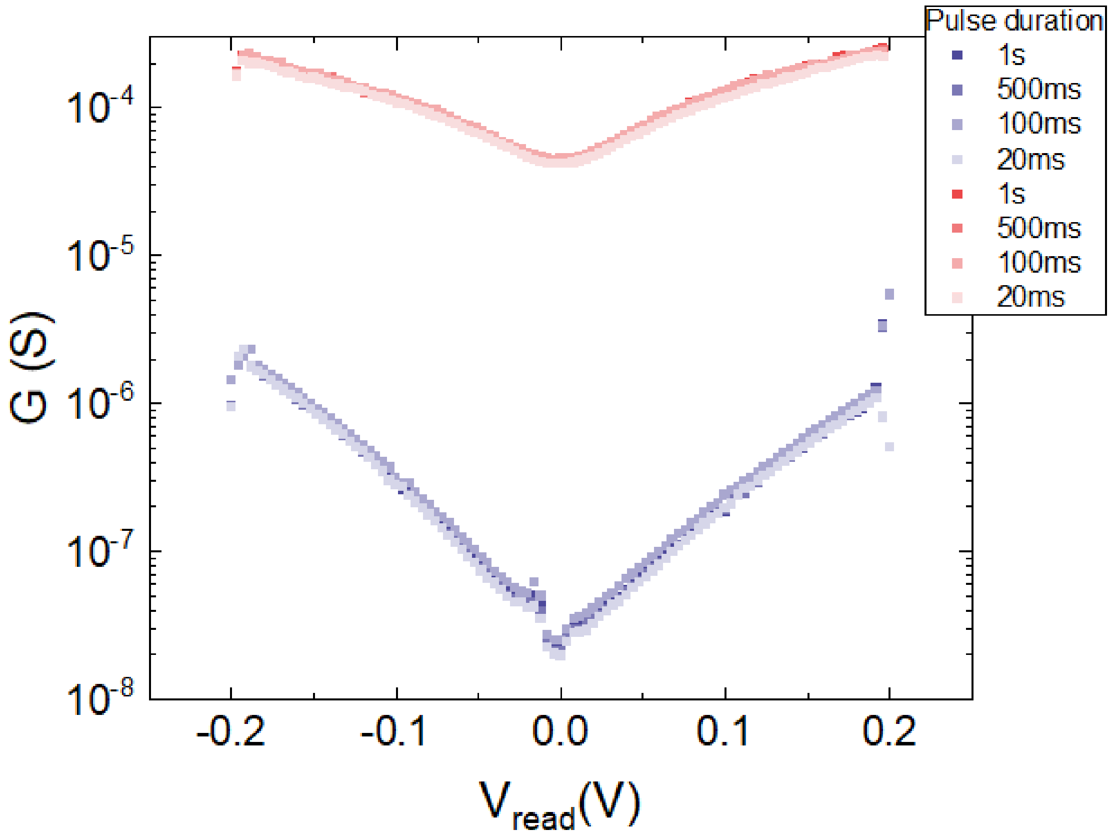

Fig. S6: Differential conductance *vs.* bias $G(V_{read})$ in the ON state (reddish curves) and OFF state (blueish curves), set by applying writing voltages pulses of amplitude V_{write}=1.9 V and V_{write}=-1.55 V respectively. Measurements were carried at 100 K. The V_{write} pulses' duration was varied between 1s and 20 ms (see figure legend). Notice that the ON/OFF conductance levels (and consequently the conductance switching amplitude) remains essentially the same regardless of the pulse duration.
